# Longitudinal relationship between posttraumatic growth and distress in lung cancer patients during neoadjuvant immunotherapy

**DOI:** 10.1016/j.ijchp.2025.100549

**Published:** 2025-02-01

**Authors:** Qiao Chu, Fenghuan Sun, Xinsheng Zhu, Haoran Xia, Dongliang Bian, Gan He, Jinhuan Yang, Peng Zhang, Yaping He

**Affiliations:** a School of Public Health, Shanghai Jiao Tong University School of Medicine, No.227 South Chongqing Rd, Shanghai 200025, China; b Department of Thoracic Surgery, Shanghai Pulmonary Hospital, School of Medicine, Tongji University, Shanghai, 200433, China; c Center for Health Technology Assessment, Shanghai Jiao Tong University China Hospital Development Institute, Shanghai Jiao Tong University, No.227 South Chongqing Rd, Shanghai 200025, China

**Keywords:** Distress, Longitudinal relationship, Lung cancer, Neoadjuvant immunotherapy, Posttraumatic growth

## Abstract

**Purpose:**

Posttraumatic growth (PTG) has been recognized as beneficial for the emotional well-being of cancer patients. However, the longitudinal relationship between PTG and emotional distress remains unclear and has rarely been investigated among patients undergoing neoadjuvant therapy. We investigated the linear and quadratic longitudinal associations between distress (depression, anxiety, and negative affect) and PTG in lung cancer patients undergoing neoadjuvant immunotherapy. We also tested individual variations in the longitudinal associations.

**Methods:**

Data were pooled from three clinical trials (*n* = 231) evaluating the efficacy of neoadjuvant immunotherapy in lung cancer patients. At the beginning of each treatment cycle, patients completed questionnaires assessing PTG and distress. Cross-lagged panel analysis was used to evaluate longitudinal associations, and multi-group structural equation modeling was conducted to examine individual variations in these relationships.

**Results:**

A unidirectional linear relationship was observed, with lower levels of distress predicting greater PTG over time. The impact of anxiety on PTG was more pronounced in patients with higher education or lower financial burdens, while the effect of negative affect was more salient in older patients. No significant quadratic effects of distress on PTG were observed.

**Conclusions:**

Lower emotional distress may facilitate the development of PTG over time. The longitudinal effect of distress on PTG varied on age, education, and financial burdens.

**Implications:**

Psychosocial interventions to promote PTG may be more effective by incorporating stress management and emotion regulation strategies, and need to be tailored to patients’ socioeconomic characteristics.

## Introduction

Being diagnosed with lung cancer is a traumatic event that can shatter an individual's fundamental beliefs and worldviews, leading to severe emotional distress (Tedeschi & Calhoun, 1995, 2004). However, research has documented the potential for posttraumatic growth (PTG) as patients cope with cancer (Casellas-Grau, Ochoa & Ruini, 2017; Wang, Chen, Zhou, Loke & Li, 2023). This positive psychological growth can manifest in various domains, such as a deeper appreciation for life, changes in life priorities, improved relationships with others, enhanced spirituality, a stronger sense of personal strength, and finding new possibilities. PTG has long been recognized as beneficial for individuals’ adjustment and well-being (Liu, Doege, Thong & Arndt, 2020). As a result, a growing number of interventions have been developed to promote PTG in cancer patients (Choi et al., 2022; Wang, Chen, Zhou, Lin et al., 2023), with the expectation that enhanced PTG will help alleviate patients’ emotional distress. However, the literature has no consensus regarding the longitudinal relationship between distress and PTG in cancer. Specifically, it remains unclear whether the development of PTG reduces cancer-related distress or if lower levels of distress foster the improvement of PTG.

Current literature presents mixed findings regarding whether distress impedes or facilitates PTG (Casellas-Grau et al., 2017). Tedeschi and Calhoun's functional-descriptive model suggests that emotional distress is a crucial driver for the development of PTG (Tedeschi & Calhoun, 2004). According to this model, emotional distress triggers cognitive reappraisal of the traumatic experience, driving patients to find personally meaningful interpretations of their cancer experience, thereby fostering psychological growth. Supporting this theory, some studies have shown that distress predicts higher levels of PTG (McDonough, Sabiston & Wrosch, 2014; Romeo et al., 2020). Conversely, other researchers have argued that severe distress in cancer patients may disrupt cognitive reappraisal and meaning-making processes, thereby impeding the development of PTG. Consistent with this perspective, some longitudinal studies have found that lower levels of distress are associated with higher levels of PTG over time (Holtmaat, van der Spek, Cuijpers, Leemans & Verdonck-de Leeuw, 2017; Nik Jaafar et al., 2022). Furthermore, a few studies on individuals who have experienced other types of trauma have observed a curvilinear relationship between stress and PTG (McCaslin et al., 2009; Shakespeare-Finch & Lurie-Beck, 2014). Specifically, a moderate level of stress may motivate individuals to seek meaning and facilitate PTG. In contrast, severe stress may hinder PTG by impairing an individual's ability to engage in adaptive cognitive reappraisal.

Empirical research on the longitudinal impact of PTG on distress among cancer patients remains limited, and the few existing studies have yielded inconsistent findings. For instance, in a longitudinal observational study, Groarke et al. (2017) demonstrated that PTG measured at six months post-diagnosis predicted lower stress levels at 12-month and 18-month follow-ups among breast cancer survivors. Additionally, interventions aimed at fostering psychological growth or meaning-making among cancer patients have shown benefits in reducing distress, providing indirect evidence for the potential longitudinal impact of PTG in alleviating distress. For example, Choi et al. (2022) implemented a PTG intervention program among 74 breast cancer patients, incorporating writing disclosure, self-reflection, and peer support groups. Their results indicated that participants in the intervention group experienced significantly lower levels of anxiety and depression compared to those in the control group. Similarly, in a randomized controlled trial involving cancer patients who had completed primary treatment, van der Spek et al. (2017) found that meaning-centered group psychotherapy—focused on deriving meaning from the cancer experience—was associated with greater reductions in depression compared to both supportive group psychotherapy without meaning-related topics and a care-as-usual group. However, contrasting findings have emerged from other longitudinal observational studies. For example, research involving patients with breast, lung, or colorectal cancer failed to identify a significant predictive relationship between PTG and subsequent emotional distress at follow-up assessments (Tomich & Helgeson, 2012; Wang, Chang, Chen, Chen & Hsu, 2014).

Several plausible reasons may explain these inconsistent findings. First, different types of distress may uniquely influence individuals’ cognitive reappraisal and meaning-making processes, leading to varied associations with PTG (Groarke et al., 2017). Second, individuals from diverse socioeconomic backgrounds may interpret distress and traumatic experiences differently (Wu et al., 2019), potentially altering the relationship between distress and PTG. Third, cancer patients at different stages of treatment and recovery may appraise distress and trauma in distinct ways, which could modify the relationship between distress and PTG. Therefore, it is essential to investigate further the longitudinal association between PTG and various types of distress across different cancer patient populations and explore how individual differences may impact this relationship.

Theoretical frameworks of PTG suggest that PTG emerges from an individual's cognitive coping, psychosocial adaptation, and search for meaning, processes that typically require a significant period after the trauma (Janoff-Bulman, Frantz, Power, & Brewin, 1997; Tedeschi & Calhoun, 2004). Consequently, most studies on cancer-related PTG have focused on cancer patients who have completed major treatment or are months or years into their treatment (Liu et al., 2020). To date, no research has explored the relationship between distress and PTG in patients undergoing neoadjuvant immunotherapy. However, it is important to note that cancer patients undergoing neoadjuvant therapy face unique stress-coping challenges, and the relationship between distress and PTG in this group may differ from that in patients receiving systemic treatment or those in the post-treatment recovery stage. Neoadjuvant immunotherapy is a relatively novel treatment strategy involving administering immunotherapy agents before surgery to reduce the size of cancerous tumors and improve surgical outcomes. This approach offers an early opportunity to target micrometastatic disease and strengthen the patient's immune system, potentially lowering the risk of recurrence after surgery (Topalian, Taube & Pardoll, 2020; Uprety, Mandrekar, Wigle, Roden & Adjei, 2020). Neoadjuvant immunotherapy typically involves several 21-day treatment cycles before surgery, making this “waiting period” particularly challenging for patients. During this time, patients may experience heightened stress from various sources. First, because neoadjuvant immunotherapy is a precursor to surgery, patients may feel anxious about the effectiveness of the therapy, which directly influences their eligibility for surgery. Second, unlike patients receiving systemic treatment, patients undergoing neoadjuvant therapy may face significant concerns about the anticipated success of surgery and the associated risks. Furthermore, these feelings of uncertainty are frequently compounded by increased worries about potential disease progression during the “waiting period”.

Despite the enhanced stress, this relatively shorter and emotionally intense treatment phase may also present opportunities for rapid psychological growth and adaptation. For patients with large tumors or tumors in difficult-to-reach locations, neoadjuvant therapy has the potential to improve the likelihood of complete surgical resection and reduce the risk of tumor recurrence. This anticipation may foster personal strength to overcome acute stress and facilitate a sense of hope and empowerment as patients take active steps toward recovery and new possibilities. Understanding the longitudinal relationship between distress and PTG among patients undergoing neoadjuvant immunotherapy provides crucial insights into stress-coping mechanisms for this growing but understudied population, and informs tailored interventions that promote PTG and adjustment in these patients.

The aim of this study is twofold. First, it seeks to test both linear and quadratic longitudinal associations between different types of distress (anxiety, depression, and general negative affect) and PTG among lung cancer patients undergoing neoadjuvant immunotherapy. For the linear associations, we also aim to examine all possible directions of the longitudinal relationship between distress and PTG. Second, the study explores whether this relationship varies based on patients’ age, educational level, and financial burden.

## Method

This study is based on pooled data from three single-arm clinical trials that evaluated the efficacy of neoadjuvant chemoimmunotherapy for patients with non-small cell lung cancer (NSCLC) (trial registration numbers: ChiCTR1900023758, ChiCTR1900024014, and NCT04379739). Data were collected from August 1, 2019, and October 31, 2022. Participants were recruited at a specialized tertiary hospital, which is renowned for its expertise in lung cancer research and treatment, and attracts patients from across the country. The authors assert that all procedures contributing to this work comply with the ethical standards of the relevant national and institutional committees on human experimentation and with the Helsinki Declaration of 1975, as revised in 2008. Ethical approval for the three trials was granted by the relevant ethics committee (protocol numbers: 19216XW, 19217XW, and 19218XW), and all enrolled patients provided written informed consent.

The detailed inclusion and exclusion criteria for each trial were reported in previously published works (Xia et al., 2024; Zhang et al., 2022; Zhu et al., 2022). The shared inclusion criteria across the three trials were: a) age 18 years or older; b) histologically or cytologically confirmed NSCLC; c) an Eastern Cooperative Oncology Group (ECOG) performance status score of 0 or 1; d) adequate lung function to permit surgical resection, as determined by pulmonary function tests; and e) resectable or potentially resectable tumor(s). The shared exclusion criteria included a) previous antitumor therapy, b) a history of tumors other than NSCLC, and c) known or suspected active autoimmune diseases.

All enrolled patients underwent a minimum of two 21-day treatment cycles, with the option for additional cycles as determined by a multidisciplinary team (MDT) evaluation, not exceeding four cycles. Patients’ demographic information was collected at enrollment. On day 1 of each cycle, patients completed a questionnaire assessing PTG, anxiety, depression, and negative affect (NA). Consequently, the time interval between each measurement wave was 21 days.

Initially, 271 patients were enrolled. Of these, 139 underwent surgery after various treatment cycles and did not complete subsequent questionnaires. Additionally, 40 patients did not complete any follow-up questionnaires due to death (*n* = 2), withdrawal after Cycle 1 (*n* = 9), or refusal to participate (*n* = 29). The analysis included only patients who completed at least one follow-up questionnaire. Consequently, valid questionnaire data were available for 231 patients at T1 (day 1 of Cycle 1), 224 at T2 (day 1 of Cycle 2), 116 at T3 (day 1 of Cycle 3), and 53 at T4 (day 1 of Cycle 4). To ensure an adequate sample size for estimating the cross-lagged panel models, we included data only from T1 to T3.

Monte Carlo simulation approach was utilized to conduct post-hoc power analysis. A sample size of 231 patients with 10,000 simulations yielded powers ranging from 0.82 to 0.85 for detecting a medium-sized regression coefficient of 0.20 (Cohen, 1988) between PTG and depression across the three measurement waves using a significance level of α = 0.05.

## Measurement

**Demographic and Clinical Characteristics**. Participants’ demographic characteristics included age, gender, education, marital status, and employment status. Financial burden was assessed by the patient-reported ratio of healthcare costs to annual family income (healthcare cost ratio) over the past year. According to the World Health Organization's definition of catastrophic health expenditure, the healthcare cost ratio was categorized into three levels using 10 % and 25 % as thresholds (Cylus, Thomson & Evetovits, 2018). Clinical information, including cancer stage and pre-treatment pathological diagnosis, was retrieved from medical records.

**PTG.** PTG was evaluated using the Chinese version of Posttraumatic Growth Inventory-Short Form (PTGI-SF) (Cann et al., 2010; Li, Qiao, Luan, Li & Wang, 2019). The questionnaire consists of 10 items organized into five dimensions: meaningful relationships, finding new possibilities, a sense of personal strength, spirituality, and appreciation of life. Participants rated the degree of change related to their cancer experience on a scale from 0 (no change) to 5 (a significant change). The total score was computed to represent levels of PTG. The scale showed good internal reliability across all four measurement waves in the present study, with Cronbach's α ranging from 0.86 to 0.92.

**Anxiety and depressive symptoms.** The Hospital Anxiety and Depression Scale (HADS) was used to assess anxiety and depressive symptoms (Li et al., 2016; Zigmond & Snaith, 1983). A higher total score on the anxiety or depression subscale indicates more severe symptoms. The present study demonstrated good internal reliability for the anxiety subscale, with Cronbach's a ranging from 0.85 to 0.87, and for the depression subscale, with Cronbach's α ranging from 0.72 to 0.79.

**NA**. NA was assessed using the negative affect subscale of the Positive and Negative Affect Scale-Short Form (PANAS-SF) (Watson, Clark & Tellegen, 1988). Participants rated their experiences of each negative emotional state over the past week on a scale from 1 (very slightly or not at all) to 5 (extremely). The current study demonstrated good internal reliability across all measurement waves, with Cronbach's α ranging from 0.91 to 0.92.

## Analysis strategy

Longitudinal association between PTG and emotional distress were examined using cross-lagged panel analysis (Wang & Zhang, 2023) in Mplus 8.9 (Muthén & Muthén, 1998–2017). A quadratic model was constructed to test whether the quadratic term of distress predicted PTG at subsequent waves, accounting for the linear effect of distress. Separate models were built for each distress indicator (anxiety, depression, and NA). Each model included three sets of parameters: a) Second-order auto-regressive AR(2) paths representing within-construct effects from each indicator to itself in subsequent waves; b) cross-sectional covariances between PTG, the linear term of distress, and the quadratic term of distress; and c) cross-lagged paths from the quadratic and linear terms of distress at Time *t* to PTG at Time *t* *+* *1.* If a cross-lagged path from Time *t* to Time *t* *+* *2* was statistically significant and its inclusion significantly improved model fit at α = 0.05, it was included in the model.

A similar approach was used for the linear model, but with only the linear term of distress and PTG as indicators. We tested all possible directions of the longitudinal linear associations between distress and PTG through three model series: Model 1 series, where distress predicted subsequent PTG; Model 2 series, where PTG predicted subsequent distress; and Model 3 series, where distress and PTG predicted each other at subsequent waves. To enhance model parsimony and interpretability, we aimed to simplify well-fitting models by equalizing conceptually analogous cross-lagged paths across measurement waves. For example, paths from depression to PTG were constrained to be equal from T1 to T2 and from T2 to T3, provided that this constraint did not significantly decrease the model fit.

We also conducted sensitivity analysis by controlling for the potential confounders. Bivariate correlations were examined between sample characteristics and model variables to identify relevant confounders. Models were then re-tested by controlling for the confounders.

Finally, we conducted a multi-group analysis to examine whether the longitudinal cross-lagged paths varied based on patients’ age (using a median split: age ≤ 65 years vs. age > 65 years), educational level (below senior high school vs. senior high school or higher), and levels of financial burden (healthcare cost ratio below 25 % vs. 25 % or higher). First, all cross-lagged paths were constrained to be equal across sub-groups. Second, we compared these constrained models to unconstrained models using the chi-square difference test, with a non-significant result indicating no differences in the paths across sub-groups. If the result was significant, modification indices were examined to identify specific paths that could be freed one at a time. This process continued until a non-significant result was achieved.

For all models, we used maximum likelihood estimation with standard errors and an adjusted χ² test statistic (MLR estimator) that are robust to potential non-normality and non-independence in the data (Muthén & Muthén, 1998–2017; Yuan & Bentler, 2000). Comparative Fit Index (CFI) and the Root Mean Square Error of Approximation (RMSEA) were also analyzed, with models achieving CFI > 0.95 and RMSEA < 0.05 considered well-fitting (Hu & Bentler, 1999).

Little's missing completely at random (MCAR) test is nonsignificant ($\chi^{2}$=22.55, *df* = 23, *p* = 0.488), suggesting the MCAR assumption may not be violated. Nevertheless, there was a marginally significant difference in educational level between patients who missed one or more follow-up questionnaires and those with complete data ($\chi^{2}$= 7.86, *p* = 0.049). The two groups did not show significant differences in other sample characteristics, including age, gender, marital status, healthcare cost ratio, and cancer stage (all *p* > 0.05). Therefore, to be conservative, missing at random (MAR) assumption was followed, and missing data were addressed using full information maximum likelihood (FIML) approach with robust standard errors.

## Results

### Participant characteristics

Enrolled patients were 19 to 77 years old (M = 63.17, *SD* = 8.88) and were predominantly men (88.7 %). Most patients were married (92.2 %), and 67.5 % had an education level below senior high school. Only 13.9 % of patients were employed full-time, while 50.6 % were retired. Nearly half of the patients (43.8 %) had a healthcare cost ratio above 25 %. The most common pathological type was Squamous cell carcinoma (57.5 %), with cancer stages ranging from IB to IIIC. Detailed sample characteristics are presented in Table 1.

**Table 1:** Sample characteristics at treatment initiation (N = 231).

Variable	Frequency/Mean (SD)	Percentage (*%*) a
Age(years)	63.17(8.88)	
Gender		
Male	205	88.7 %
Female	26	11.3 %
Education		
Elementary school or lower	65	28.1 %
Junior high school	91	39.4 %
Senior high school	53	22.9 %
College or higher	21	9.1 %
Marital status		
Married	213	92.2 %
Single/divorced/widowed	18	7.8 %
Employment status		
Full-time/Part-time employed	32	13.9 %
Unemployed/sick leave	66	28.6 %
Retired	117	50.6 %
Other	10	4.3 %
Healthcare-cost-to-income ratio^b^		
<10 %	59	21.7 %
10∼25 %	71	26.1 %
>25 %	119	43.8 %
Cancer stage		
I-II	43	18.6 %
IIIA	139	60.2 %
IIIB	38	16.5 %
IIIC	11	4.8 %
Pre-treatment pathological diagnosis		
Squamous cell carcinoma	133	57.6 %
Adenocarcinoma	37	16.0 %
NSCLC not otherwise specified	51	22.1 %
Other	10	4.3 %

a Percentages may not add up to 100 % because of missing data.

b According to the definition of catastrophic health expenditure proposed by the World Health Organization (WHO), the healthcare-cost-to-income ratio was divided into three groups, using 10 % and 25 % as thresholds (Cylus et al., 2018).

### Quadratic relationship model

**Fig. 1:**
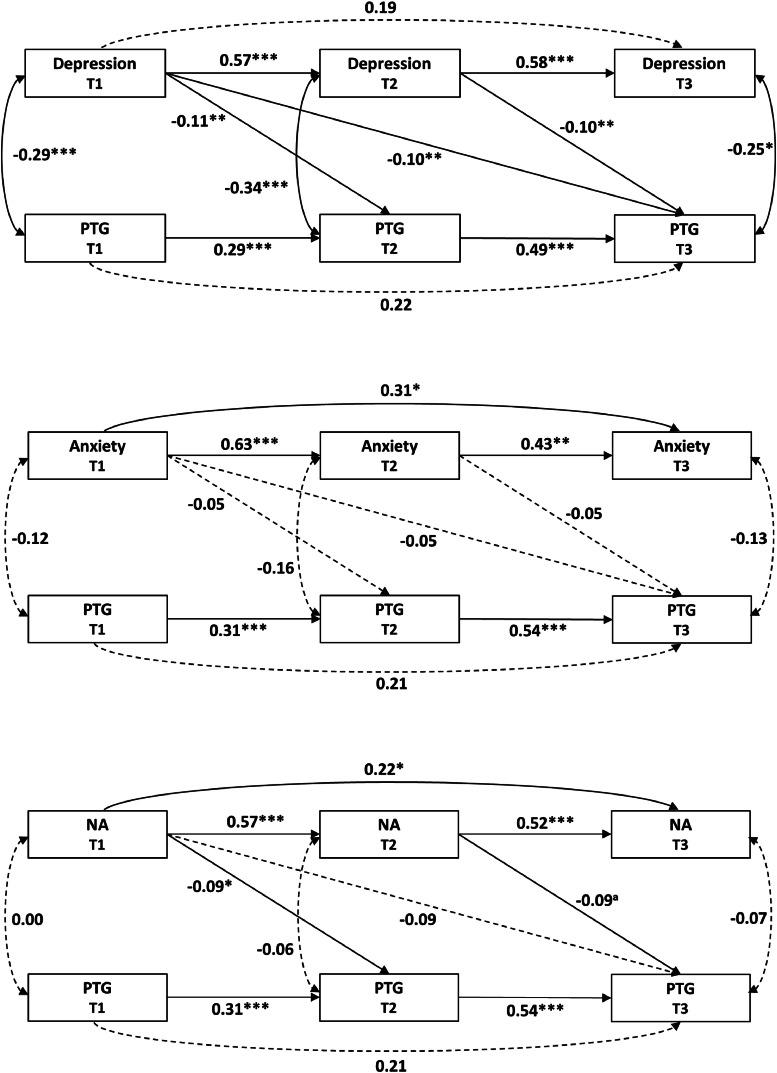
Quadratic relationship models, with distress predicting PTG. All paths shown are standardized regression coefficients. Solid lines indicate significant paths. Dashed lines indicate nonsignificant paths. *p < 0.05, **p < 0.01, ***p < 0.001.

The fit indices for all models are presented in Table 2, with the Model paths in Fig. 1. The depression-PTG quadratic model indicated a good fit; however, all quadratic paths were nonsignificant (all *p* > 0.05), while all linear paths were significant (all *p* < 0.05). The anxiety-PTG quadratic model demonstrated inadequate fit, with both quadratic and linear paths being nonsignificant (all *p* > 0.05). The NA-PTG quadratic model indicated adequate fit, but both quadratic and linear paths were nonsignificant (all *p* > 0.05). Therefore, the quadratic relationships were not supported for any distress indicator.

**Table 2:** Fit indices for quadratic and linear relationship models.

**Model description**	χ*^2^*(*df*)	*p*	CFI	RMSEA	SRMR	
**Quadratic association Model**						
Quadratic Model a. Depression^2^→PTG	13.99(16)	.600	1.000	0.000		0.036
Quadratic Model b. Anxiety^2^→PTG	23.67(16)	.097	0.965	0.046		0.063
Quadratic Model c. NA^2^→PTG	21.69(16)	.154	0.975	0.039		0.052
**Linear association model**						
**Model 1 series (distress predicting PTG)**						
Linear Model 1a. Depression → PTG	5.29(5)	.381	0.999	0.016	0.030	
Linear Model 1b. Anxiety → PTG	4.11(5)	.533	1.000	0.000	0.025	
Linear Model 1c. NA → PTG	5.66(5)	.341	0.996	0.024	0.039	
**Model 2 series (PTG predicting distress)**						
Linear Model 2a. PTG → Depression	11.63(3)	.009	0.959	0.112	0.071	
Linear Model 2b. PTG → Anxiety	2.55(3)	.467	1.000	0.000	0.040	
Linear Model 2c. PTG → NA	9.03(3)	.029	0.962	0.094	0.066	
**Model 3 series (bidirectional effects)**						
Linear Model 3a. Depression ←→ PTG	4.66(4)	.324	0.997	0.027	0.022	
Linear Model 3b. Anxiety ←→ PTG	4.08(4)	.396	1.000	0.009	0.025	
Linear Model 3c. NA ←→ PTG	4.31(4)	.365	0.998	0.019	0.022	

Note. PTG = posttraumatic growth.

### Linear relationship model

**Fig. 2:**
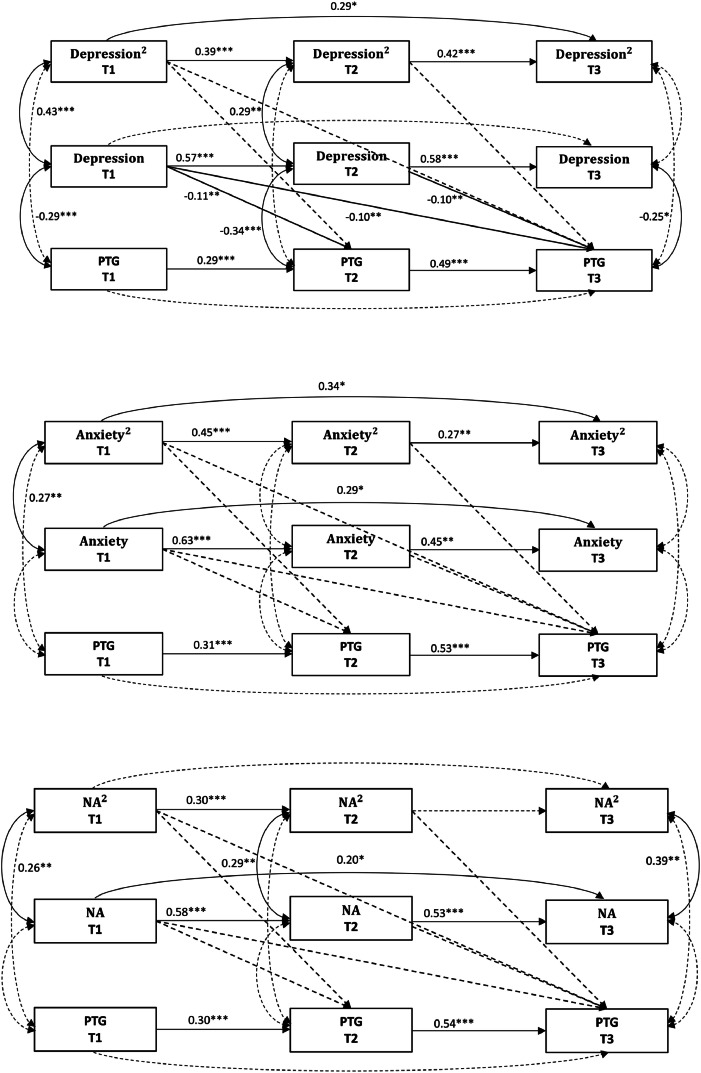
Final linear relationship models (model 1 series), with distress predicting PTG. All paths shown are standardized regression coefficients. Solid lines indicate significant paths. Dashed lines indicate nonsignificant paths. *p < 0.05, **p < 0.01, ***p < 0.001, ^a^*p* = 0.050.

**Model 1 series (distress predicting subsequent PTG).** All models showed a good fit. In the depression-PTG model, all paths from depression to PTG were significant (all *p* < 0.05), with depression being negatively associated with PTG across subsequent waves. In the anxiety-PTG model, all paths from anxiety to PTG were nonsignificant (all *p* > 0.05). For the NA-PTG model, the paths from NA to PTG ranged from significant to marginally significant (*p* = 0.041–0.053), with NA negatively associated with PTG. The model paths are presented in Fig. 2.^3^

**Model 2 series (PTG predicting subsequent distress).** The PTG-depression model showed a poor fit, with all paths from PTG to depression being nonsignificant (all *p* > 0.05). The PTG-anxiety model demonstrated a good fit, and all paths from PTG to anxiety were nonsignificant (all *p* > 0.05). The PTG-NA model also indicated poor fit, and all paths from PTG to NA were nonsignificant (all *p* > 0.05). The model paths are presented in supplementary Figure S1.

**Model 3 series (bidirectional associations).** The depression-PTG model indicated a good fit, but only the paths from depression to PTG were significant and negative (all *p* < 0.05). In contrast, all paths from PTG to depression were nonsignificant (all *p* > 0.05). The anxiety-PTG model also showed a good fit, though all cross-lagged paths were nonsignificant (all *p* > 0.05). The NA-PTG model indicated a good fit, with paths from NA to PTG ranging from significant to marginally significant (*p* = 0.041–0.052), while all paths from PTG to NA were nonsignificant (all *p* > 0.05). The model paths are presented in Supplementary Figure S2.

In summary, the linear models supported a unidirectional impact of depression and NA on subsequent PTG. The fit indices for all models are presented in Table 2.

### Sensitivity analysis

We conducted a sensitivity analysis to account for potential confounders. The results of bivariate correlations indicated that age was significantly associated with PTG at T2 (*r* = −0.21 *p* = 0.002) and T3 (*r* =−0.26, *p* = 0.005), cancer stage was significantly related to PTG at T3 (*r* = 0.19, *p* = 0.046), and surgery status was significantly associated with depression at T2 (*r* = −0.15, *p* = 0.029). No other significant correlations were found (all *p* > 0.05). Next, we re-tested the Linear relationship Model 1 series, controlling for these confounders. The results showed minimal changes in model fit indices and model path estimates. The adjusted model results are presented in Supplementary Figure S3.

### Individual variations on the linear effects of distress on PTG

We further tested whether the unidirectional linear paths from distress to PTG varied depending on age, educational level, or financial burden.

For the depression-PTG linear model, the paths from depression to PTG did not vary significantly by age, educational level, or financial burden, as indicated by nonsignificant chi-square difference tests between the constrained and unconstrained models (all *p* > 0.05).

In the anxiety-PTG linear model, the path from anxiety at T1 to PTG at T3 was significant only for patients with at least a senior high school education (standardized coefficient = −0.37, *p* = 0.001) but nonsignificant for patients with lower education levels (standardized coefficient < 0.001, *p* = 0.999) (see Supplementary Figure S4). Moreover, the path from anxiety at T2 to PTG at T3 was significant only for patients with a healthcare cost ratio below 25 % (standardized coefficient = −0.36, *p* = 0.002), compared to those with a higher financial burden (standardized coefficient = 0.15, *p* = 0.113) (see Supplementary Figure S5). There were no significant age differences in the model paths (*p* > 0.05).

For the NA-PTG linear model, the path from NA at T1 to PTG at T3 was significant only for patients older than 65 years old (standardized coefficient = −0.32, *p* = 0.009) but not significant for younger patients (standardized coefficient = −0.01, *p* = 0.936) (see Supplementary Figure S6). The model paths did not significantly vary by educational level or financial burden (*p* > 0.05).

## Discussion

Based on data from a cohort of lung cancer patients receiving neoadjuvant chemoimmunotherapy, this study yielded two key findings. First, the results supported unidirectional linear effects of distress on subsequent PTG, with lower levels of depression and NA predicting higher PTG. Second, the impact of distress on PTG significantly varied based on patients’ age, education level, and financial burden. Specifically, the effect of anxiety on PTG was more pronounced in patients with higher education levels or lower financial burdens, while the impact of NA on PTG was more pronounced in older patients.

We did not find significant quadratic effects of any indicator of distress on PTG. Quadratic (curvilinear) effects of distress on PTG have been investigated in only a limited number of studies, and are rarely tested among cancer patients (Shakespeare-Finch & Lurie-Beck, 2014). A meta-analysis on the association between PTSD and PTG suggested that the significance and magnitude of curvilinear associations vary across different trauma types (Shakespeare-Finch & Lurie-Beck, 2014). It is also likely that the presence of curvilinear associations varies depending on the types of distress being studied. For example, a cross-sectional study found significant quadratic effects of general perceived stress on PTG among breast cancer survivors, but nonsignificant quadratic effects of cancer-related worries (Coroiu, Körner, Burke, Meterissian & Sabiston, 2016). Different kinds of distress may have differential impacts on individuals’ cognitive appraisals and coping processes, thus influencing PTG in varying ways. Given the limited prior evidence among cancer patients, further research is needed to confirm these findings.

Our findings supported significant unidirectional linear effects of depression and NA on subsequent PTG throughout neoadjuvant immunotherapy for lung cancer patients. Specifically, higher levels of depression and NA predicted lower levels of subsequent PTG. These results align with previous longitudinal studies that demonstrated an inverse relationship between distress and PTG among cancer patients (Holtmaat et al., 2017; Nik Jaafar et al., 2022). These findings suggest that overwhelming distress may hinder patients’ ability to engage in cognitive reappraisal and derive meaning from their cancer experience, thereby impeding the development of PTG.

In contrast to our findings, some studies have reported a positive association or no significant association between distress and PTG in cancer patients (Casellas-Grau et al., 2017). This inconsistency may be partially attributed to differences in sample characteristics across studies. Individuals from varying socioeconomic backgrounds may appraise trauma and emotional distress differently, which can affect their processes of meaning-making and benefit-finding. Supporting this perspective, our study observed that the longitudinal effects of anxiety and NA on PTG varied according to the patient's age, education level, and financial burden. Specifically, the negative impact of anxiety on PTG was significant only among patients with higher education levels and those with lower financial burdens. Previous research has demonstrated that socioeconomic status influences individuals’ cognitive processing capacities (Elsayed, Luby & Barch, 2023; Hao & Farah, 2020). Patients with higher education levels and greater financial resources may possess greater self-awareness and higher expectations regarding their ability to manage cancer-related stress. As a result, elevated anxiety may be perceived as a significant threat to their sense of control, intensifying its negative impact on their ability to derive meaning from their cancer experience and foster PTG. In contrast, patients with lower education levels or greater financial burdens may be more preoccupied with addressing immediate and tangible concerns, limiting their capacity to engage in the deeper cognitive reappraisal processes necessary for meaningful reflection. Given this limited engagement, heightened anxiety may have a less pronounced effect on their ability to develop PTG (Keltner, Gruenfeld & Anderson, 2003). Furthermore, our study found that the negative impact of NA on PTG was significant only among patients over the age of 65, but not among younger patients. It would appear that older adults are more prone to engage in life review and existential reflection (van Rhyn, Barwick & Donelly, 2022). Consequently, high levels of NA in this context might be more strongly tied to feelings of regret and loss, which could impede the process of deriving meaning or experiencing growth from the cancer experience.

We did not observe a significant impact of PTG on subsequent distress. This finding aligns with previous studies on patients with lung, colorectal, or breast cancer (Tomich & Helgeson, 2012; Wang et al., 2014). Nevertheless, a study found that PTG at six months after breast cancer diagnosis predicted lower levels of stress at 12-month and 18-month follow-ups (Groarke et al., 2017). When interpreting these inconsistent findings, one could not rule out the possibility that the effects of PTG on distress may not become evident until a more extended period has passed following the trauma. Since patients in our study were followed for a maximum of six weeks after cancer diagnosis, further research is needed to determine whether the impact of PTG on distress would become more salient over extended follow-up periods.

Another critical factor that may contribute to the inconsistent findings regarding the longitudinal relationship between PTG and emotional distress is the role of posttraumatic depreciation (PTD)——the negative psychological changes individuals may experience following trauma (Baker, Kelly, Calhoun, Cann & Tedeschi, 2008). Studies have suggested that PTG and PTD are independent constructs, and individuals can experience both simultaneously when coping with trauma (Taku et al., 2021; Zięba, Wiecheć, Biegańska-Banaś & Mieleszczenko-Kowszewicz, 2019). Researchers have pointed out that assessing both positive and negative changes may reduce the likelihood of a positive response bias in assessing PTG, and provides a more comprehensive understanding of individuals’ posttraumatic experience (Baker et al., 2008; Gower, Pham, Jouriles, Rosenfield & Bowen, 2022). Notably, studies have demonstrated that both PTG and PTD are associated with emotional distress (Kunz, Joseph, Geyh & Peter, 2017; Michélsen, Therup-Svedenlöf, Backheden & Schulman, 2017). Furthermore, PTD has been found to moderate the association between PTG and emotional distress, as observed in spinal cord injury patients (Kunz et al., 2017). In light of this, it is likely that the longitudinal association between emotional distress and PTG observed in our study may also be moderated by patients’ levels of PTD. Future studies should seek to confirm our findings using measures that capture both positive and negative posttraumatic changes, such as the Psychological Well-Being-Posttraumatic Changes Questionnaire (PWB-PTCQ) (Joseph et al., 2012) and the Posttraumatic Growth and Posttraumatic Depreciation Inventory-Expanded version (PTGDI-X) (Taku et al., 2021).

### Clinical and psychological implications

PTG has long been regarded as a contributor to the well-being of cancer patients, with interventions designed to enhance PTG often expected to also alleviate emotional distress (Choi et al., 2022; Wang, Chen, Zhou, Lin et al., 2023). However, our findings suggest that, in this specific cohort of lung cancer patients undergoing neoadjuvant immunotherapy, the longitudinal relationship between PTG and emotional distress may be more complex than previously assumed. Specifically, lower levels of emotional distress appear to precede the development of PTG over time, rather than PTG leading to reductions in distress. These results imply that interventions focused solely on promoting PTG may not be sufficient to mitigate emotional distress in this population. Therefore, psychosocial interventions aimed at fostering PTG could be more effective by incorporating components such as stress management and emotion regulation to address distress comprehensively.

Additionally, we found that the longitudinal impact of emotional distress on PTG was more pronounced among patients with higher education levels, lower financial burdens, and relatively older age. This finding suggests that in clinical trial settings, interventions aimed at fostering PTG may need to be tailored to patients’ socioeconomic characteristics. Research indicates that cancer patients enrolled in clinical trials are at high risk of experiencing financial burdens (Perni et al., 2022; Winkfield et al., 2018). In our study, although patients did not bear the direct cost of immunotherapy, they might still face significant financial strain from ancillary costs (e.g., transportation, lodging near the hospital, and nutritional needs) and indirect costs (e.g., patient or caregiver unemployment, reduced work time, and lost wages). Additionally, clinical trial participation often involves more frequent clinic visits and longer travel distances, which can further exacerbate financial challenges (Nipp, Hong & Paskett, 2019). These financial burdens may interfere with the cognitive processes necessary for translating emotional distress into psychological growth. Thus, for patients experiencing relatively greater financial strain, interventions may benefit from integrated psychosocial and financial counseling, as well as practical support services, to help them better manage stress and facilitate PTG.

### Limitations

This study has several limitations. First, our study participants were clinical trial patients, who may experience different financial challenges compared to those outside the trial setting. As a result, our findings may not be fully generalizable to broader patient populations. Future research is needed to confirm these findings in more diverse and financially varied populations. Second, there is an underrepresentation of females in our sample. This disparity may be attributed to the fact that squamous cell carcinomas, which constitute the majority of pathological diagnoses in our study, are more prevalent in males (Caldarella et al., 2007; Visbal et al., 2004). The high prevalence of squamous cell carcinomas in our study could be related to their typical location near vessels and bronchi, making surgical intervention more challenging and thus making these patients more likely candidates for neoadjuvant therapy. Future research should aim to confirm these findings in a more gender-balanced sample. Third, although the Posttraumatic Growth Inventory (PTGI) is widely used to assess perceived PTG, researchers have raised concerns about its potential to introduce positive response bias, as it focuses exclusively on positive posttraumatic changes. Future studies could benefit from using instruments that allow for the reporting of both positive and negative changes. Fourth, this study was conducted within a single center. Future studies are needed to determine whether these findings can be generalized to other cancer patient cohorts. Fifth, the observed effect sizes were generally small, indicating that other factors may influence PTG. Further research is necessary to identify additional factors affecting patients’ PTG and to explore potential interactions among these factors. Finally, given that cancer patients undergoing neoadjuvant therapy face unique stress-coping challenges, the association between PTG and distress in this population may differ from that of patients receiving systemic treatment or those in the post-treatment recovery stage. As a result, the findings of this study may not be generalizable to other patient populations.

## Conclusion

In conclusion, our study highlights the unidirectional linear impact of distress on PTG among lung cancer patients undergoing neoadjuvant immunotherapy, with lower levels of distress predicting greater PTG over time. Moreover, our findings underscore the importance of tailoring interventions to address the needs of patients with different socioeconomic backgrounds to enhance PTG effectively.

## Funding

This work was supported by the 10.13039/501100001809National Natural Science Foundation of China [grant numbers: 72004133, 71874111]; The China Medical Board Open Competition Grants Program [grant number: 22-479]; Innovation Program of Shanghai Municipal Education Commission [grant number: 2023ZKZD33]; and Clinical Research Foundation of Shanghai Pulmonary Hospital [grant number: FKLY20004].

## Declaration of competing interest

The authors declare that they have no known competing financial interests or personal relationships that could have appeared to influence the work reported in this paper.

## Appendix Supplementary materials

Image, application 1

## Footnotes

3 We also tested an alternative model that includes anxiety, depression, and NA as simultaneous predictors of PTG at subsequent waves. The results of this model are presented in Supplementary Figure S7.
