# Supplementary material for: Longitudinal relationship between posttraumatic growth and distress in lung cancer patients during neoadjuvant immunotherapy
Source: Int J Clin Health Psychol. 2025 Feb 1;25(1):100549. doi: 10.1016/j.ijchp.2025.100549 (PMC11840545; doi:10.1016/j.ijchp.2025.100549)
Supplement: Supplementary file 1 [file mmc1.docx]

**Longitudinal Relationship Between Posttraumatic Growth and Distress in Lung Cancer Patients**

**During Neoadjuvant Immunotherapy**

**Online Supplement**


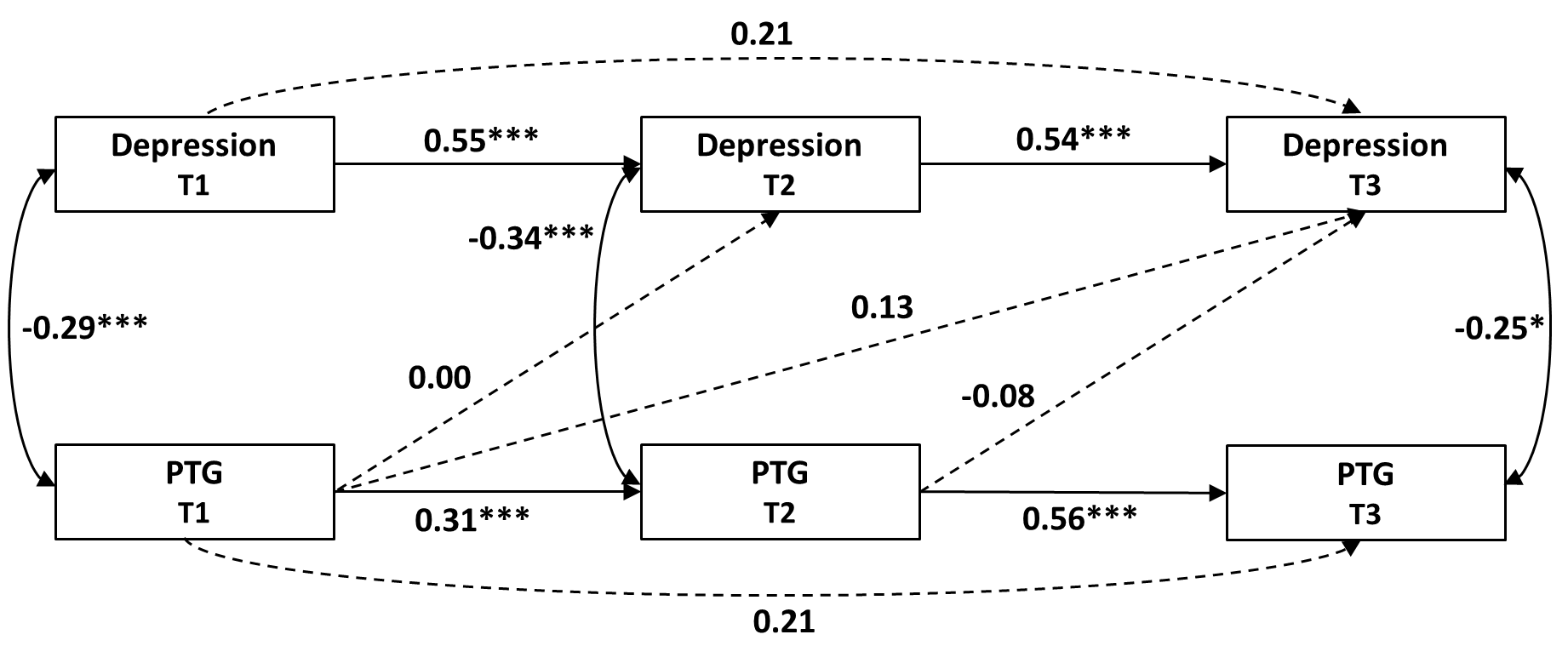


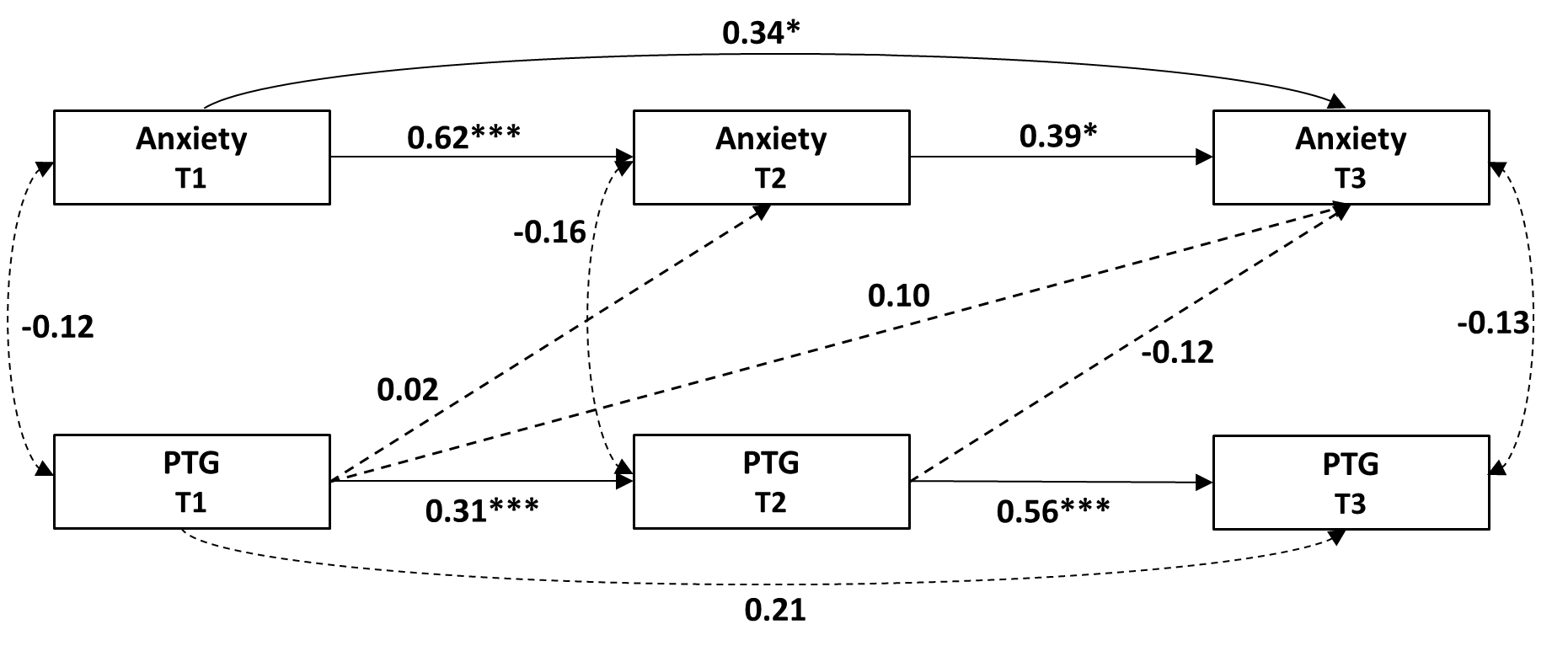


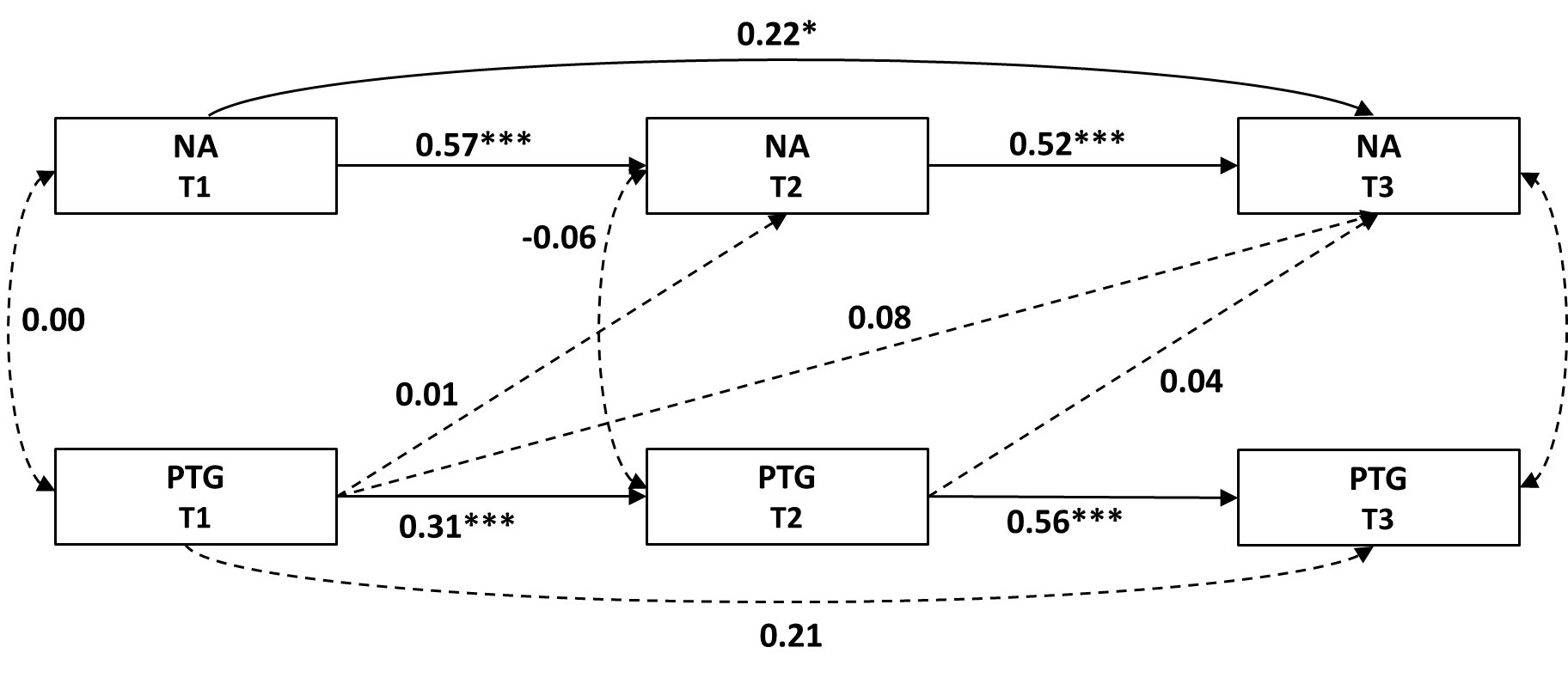


**Figure S1.** Model 2 series, with PTG predicting distress. All paths shown are standardized regression coefficients. Solid lines indicate significant paths. Dashed lines indicate nonsignificant paths. PTG = posttraumatic growth. NA = negative affect. **p* < 0.05, ***p* < 0.01, ****p* < 0.001

**
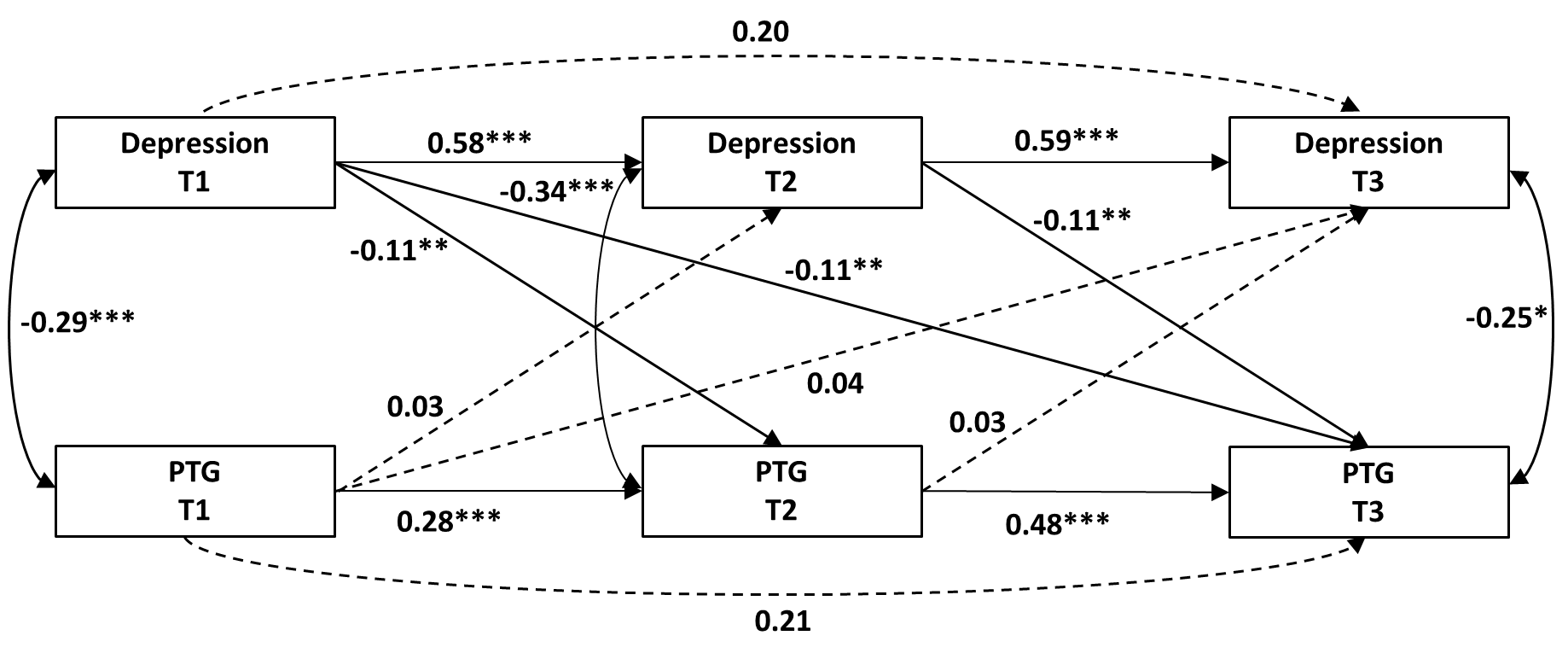
**

**
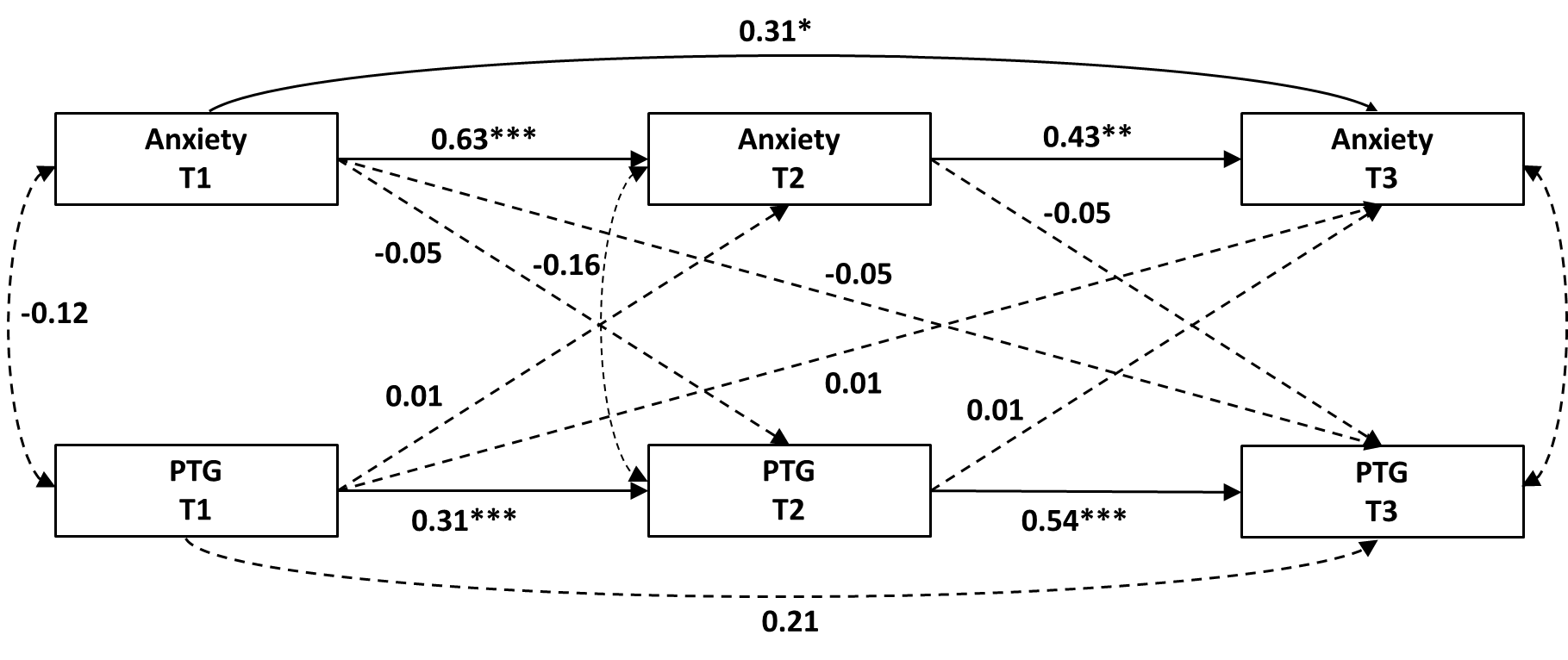
**

**
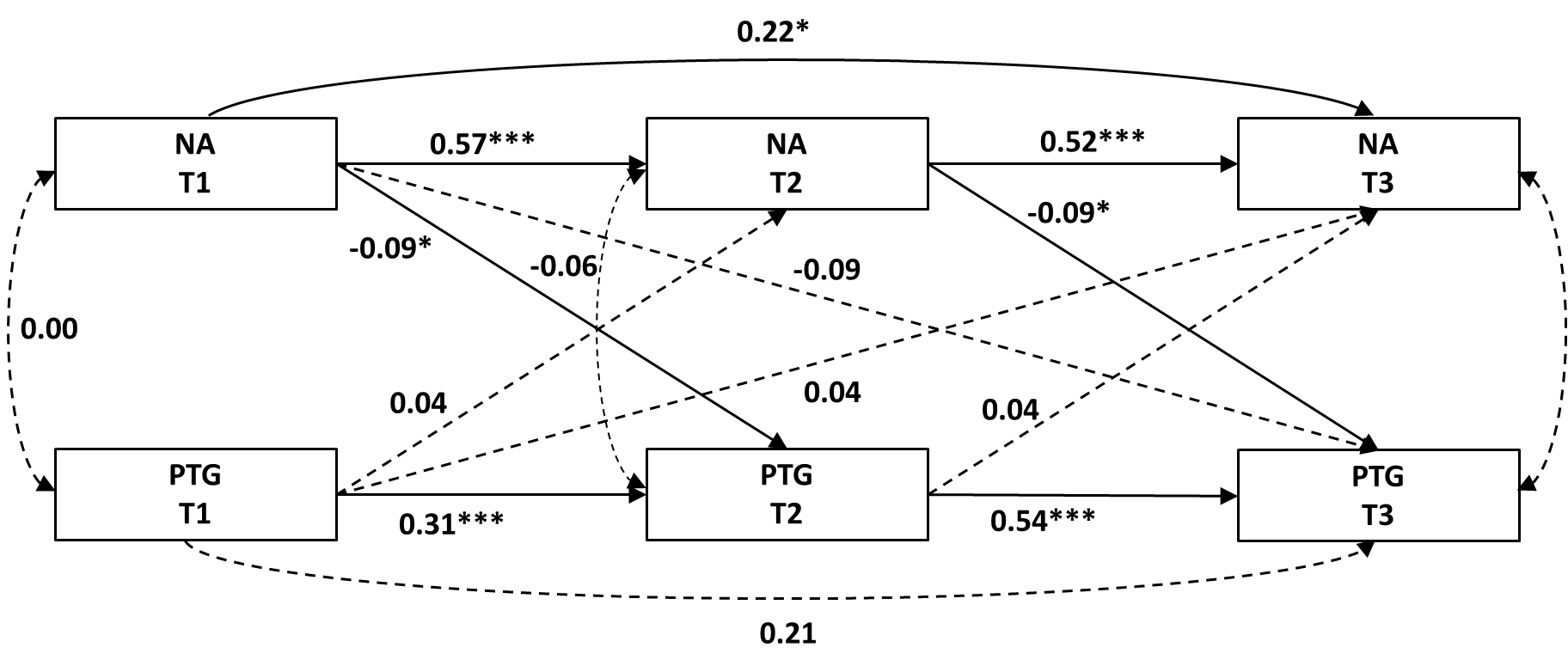
**

**Figure S2.** Model 3 series, with distress and PTG predicting each other at subsequent waves. All paths shown are standardized regression coefficients. Solid lines indicate significant paths. Dashed lines indicate nonsignificant paths. PTG = posttraumatic growth. NA = negative affect.

**p* < 0.05, ***p* < 0.01, ****p* < 0.001


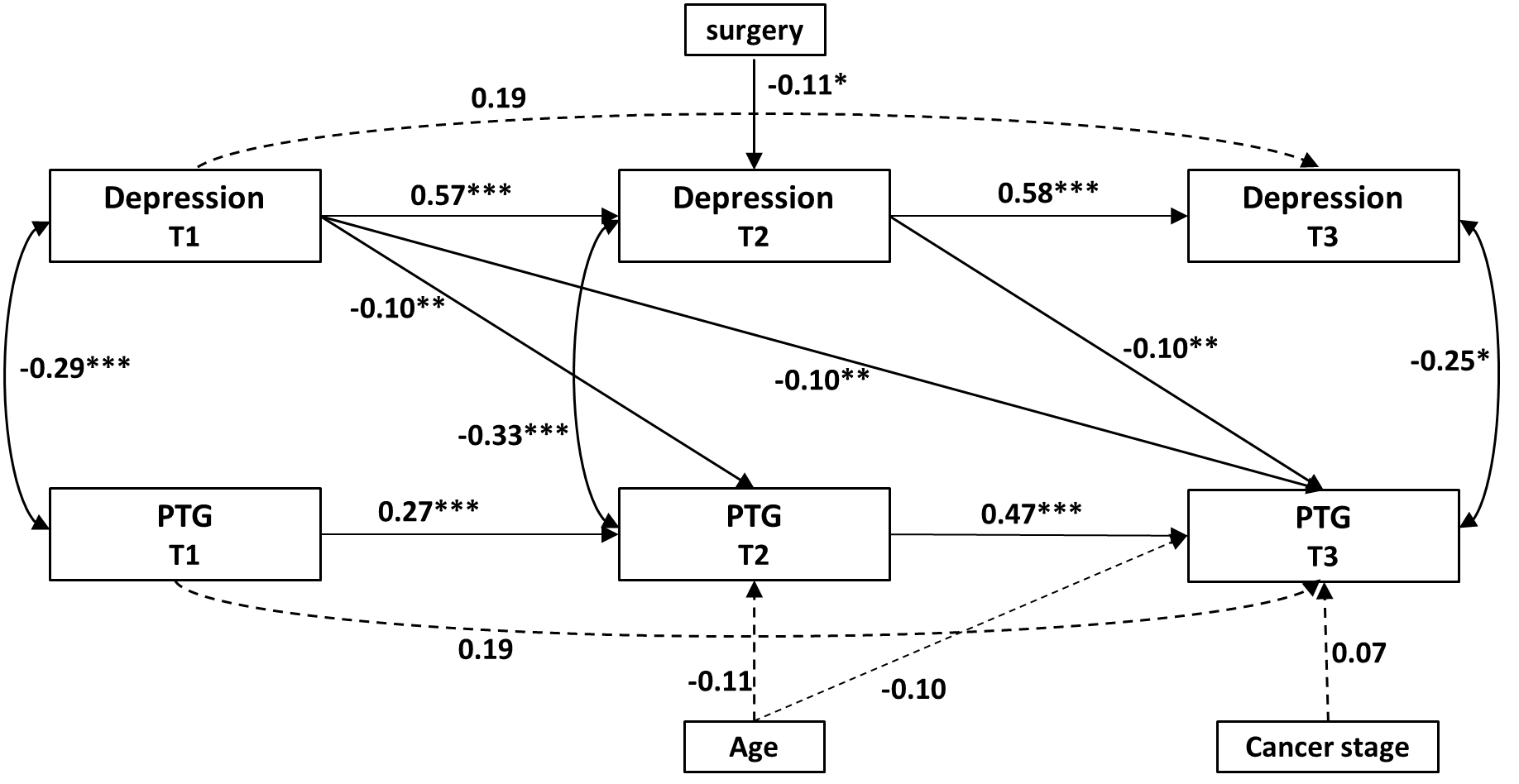


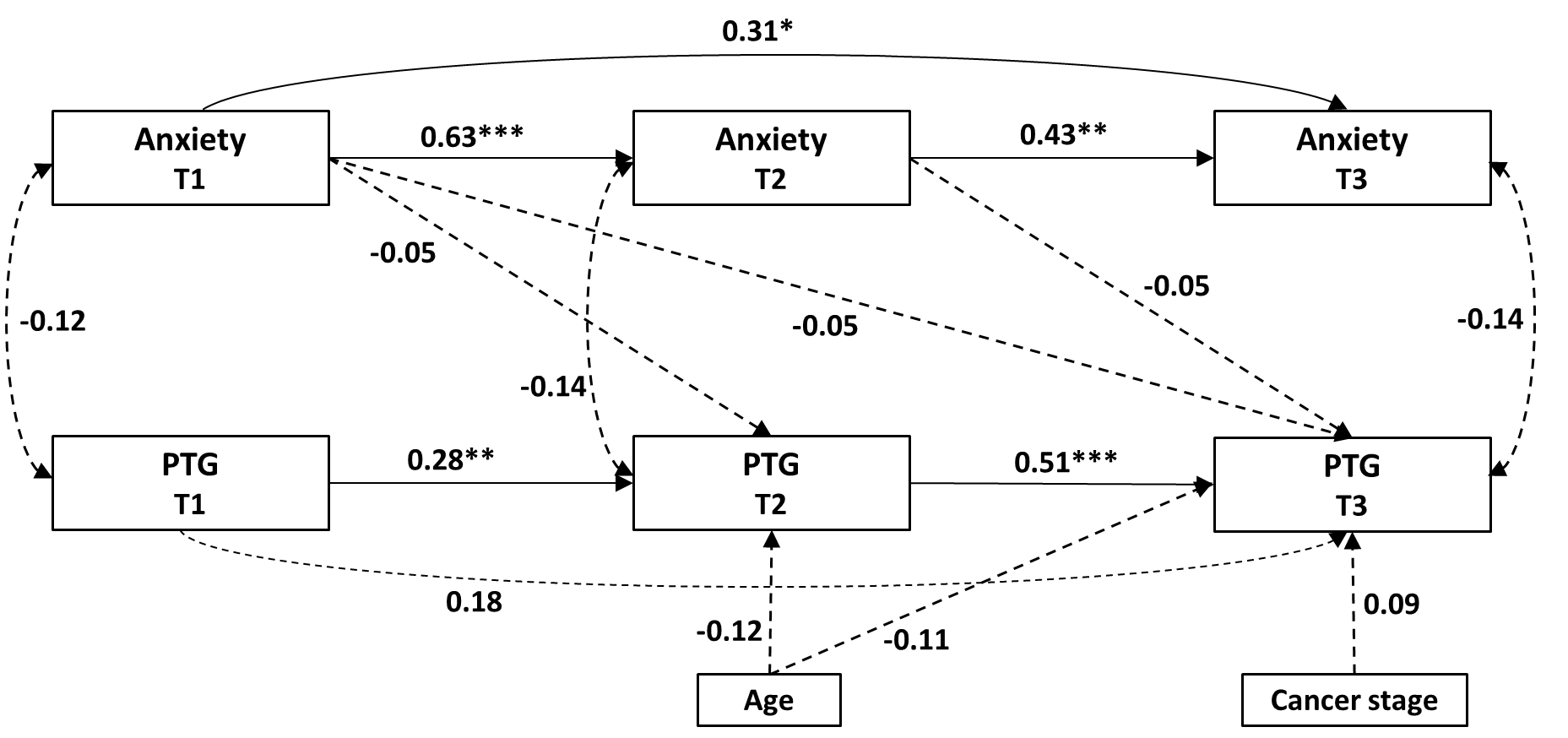


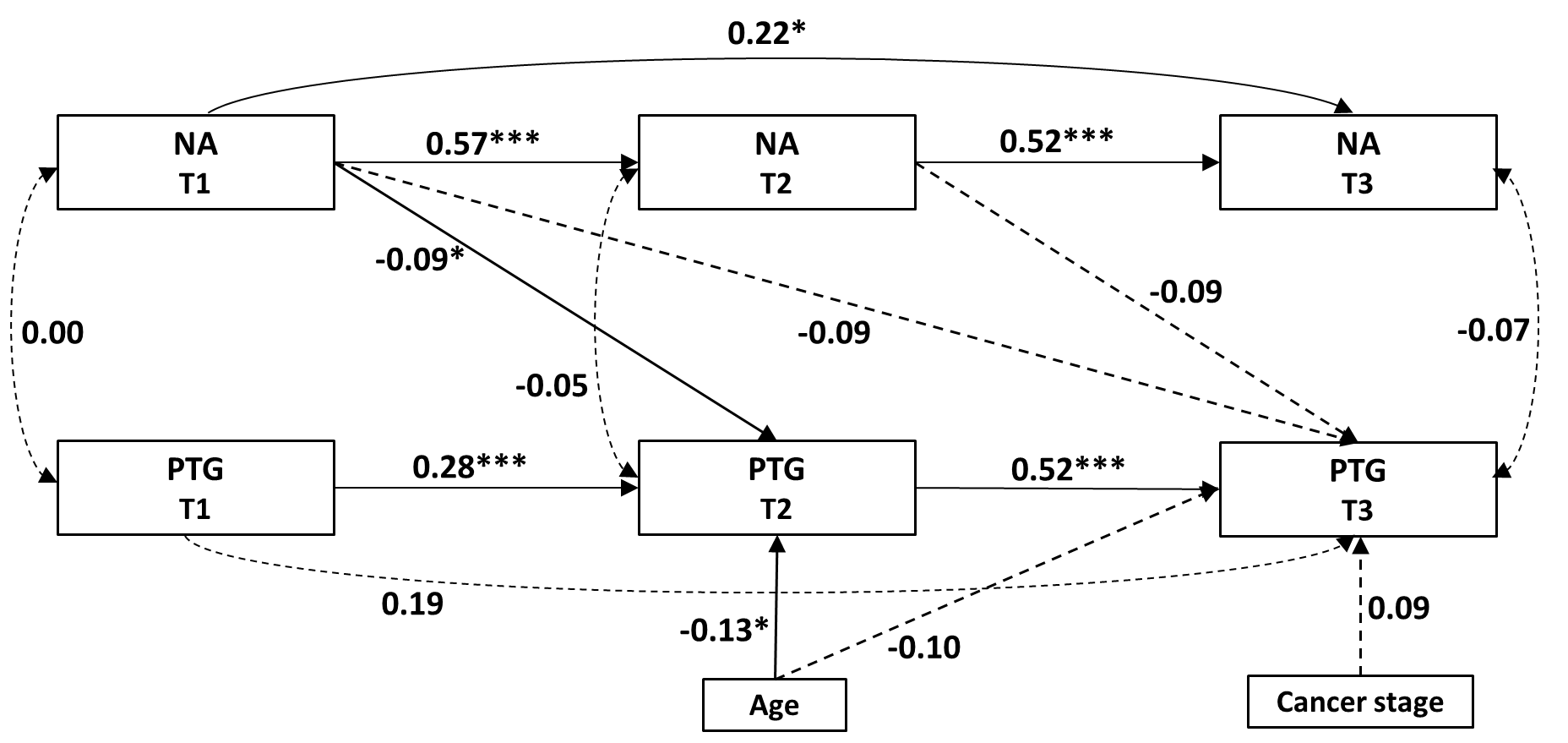


**Figure S3.** Sensitivity analysis for the Linear relationship model 1 series, controlling for relevant confounders (age, cancer stage, and surgery status). Solid lines indicate significant paths. Dashed lines indicate nonsignificant paths. PTG = posttraumatic growth. NA = negative affect.

**p* < 0.05, ***p* < 0.01, ****p* < 0.001


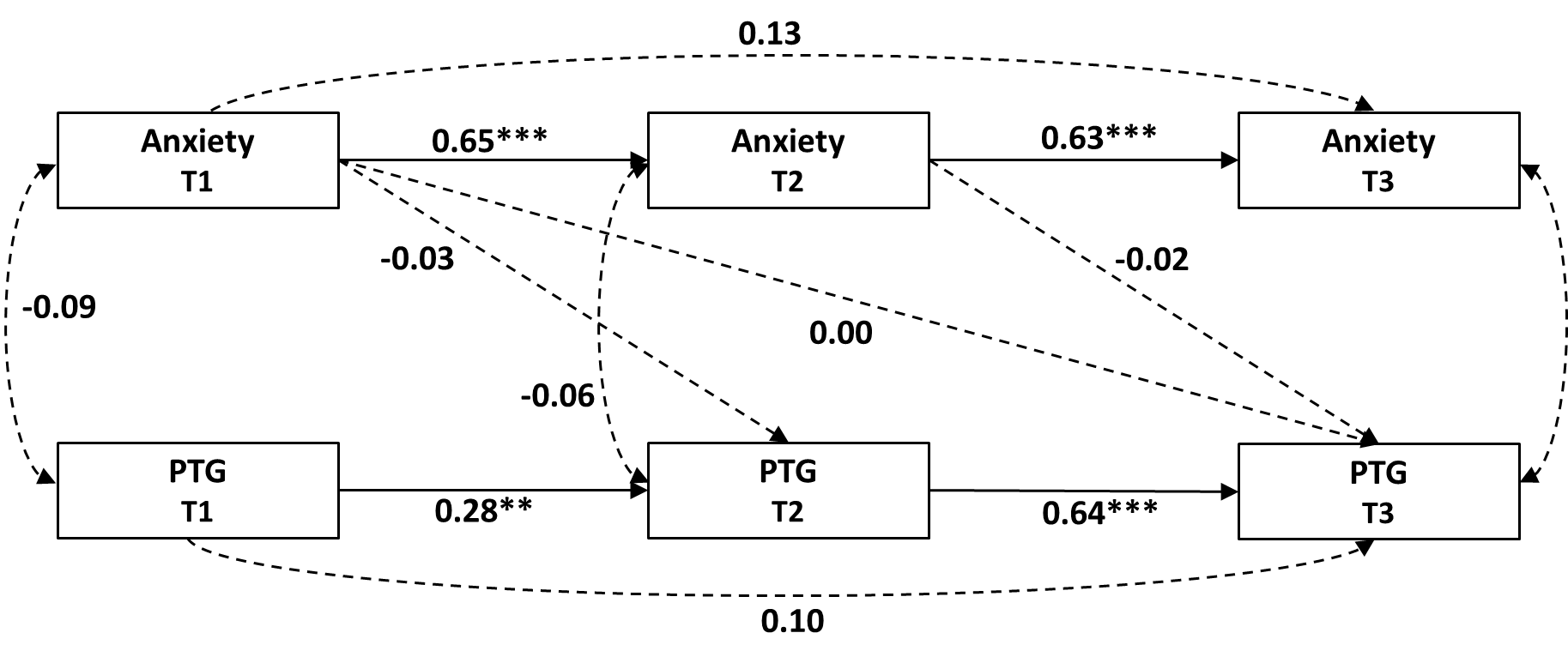


**Patients with lower education level**

**
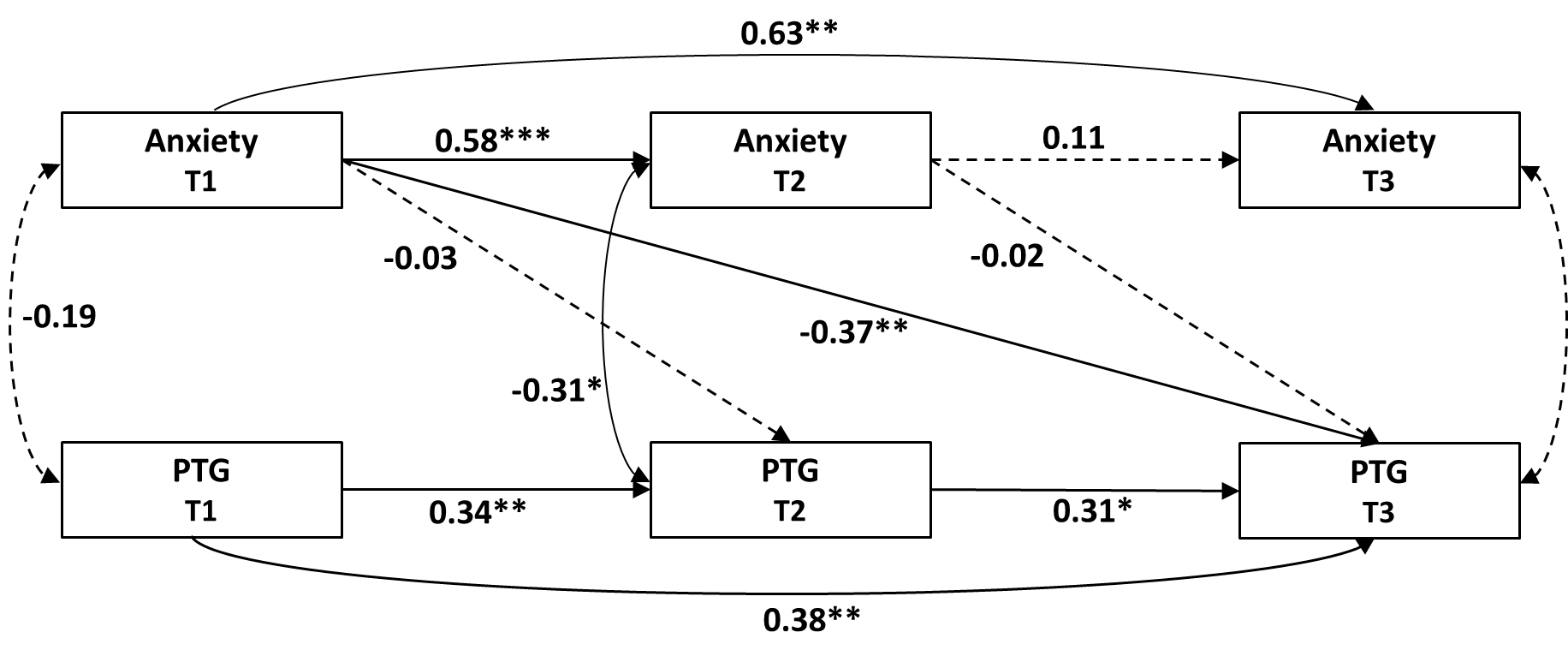
**

**Patients with higher education level**

**Figure S4.** The linear impact of anxiety on PTG, varied by education level (below senior high school vs. senior high school or higher). All paths shown are standardized regression coefficients. Solid lines indicate significant paths. Dashed lines indicate nonsignificant paths. PTG = posttraumatic growth

**p* < 0.05, ^**^*p* < 0.01, ^***^*p* < 0.001

**
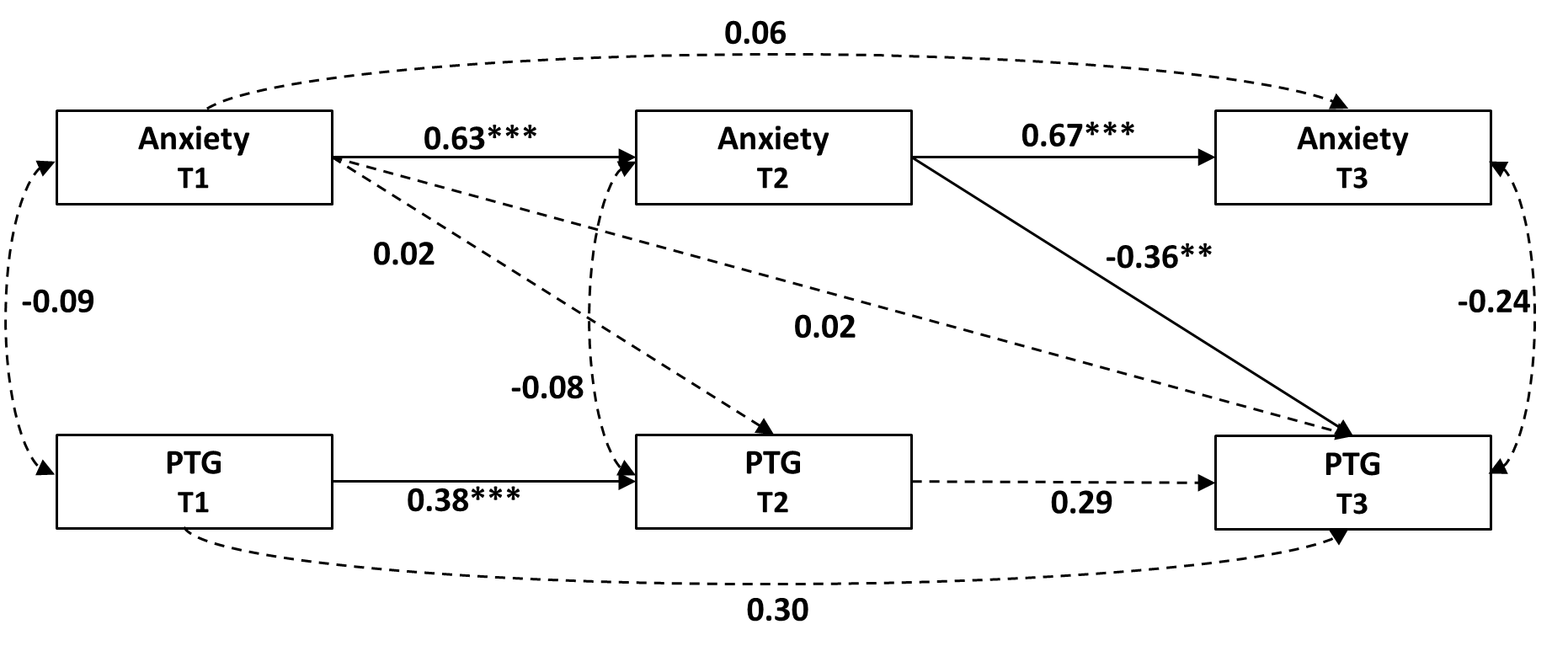
**

**Patients with lower financial burden**

**
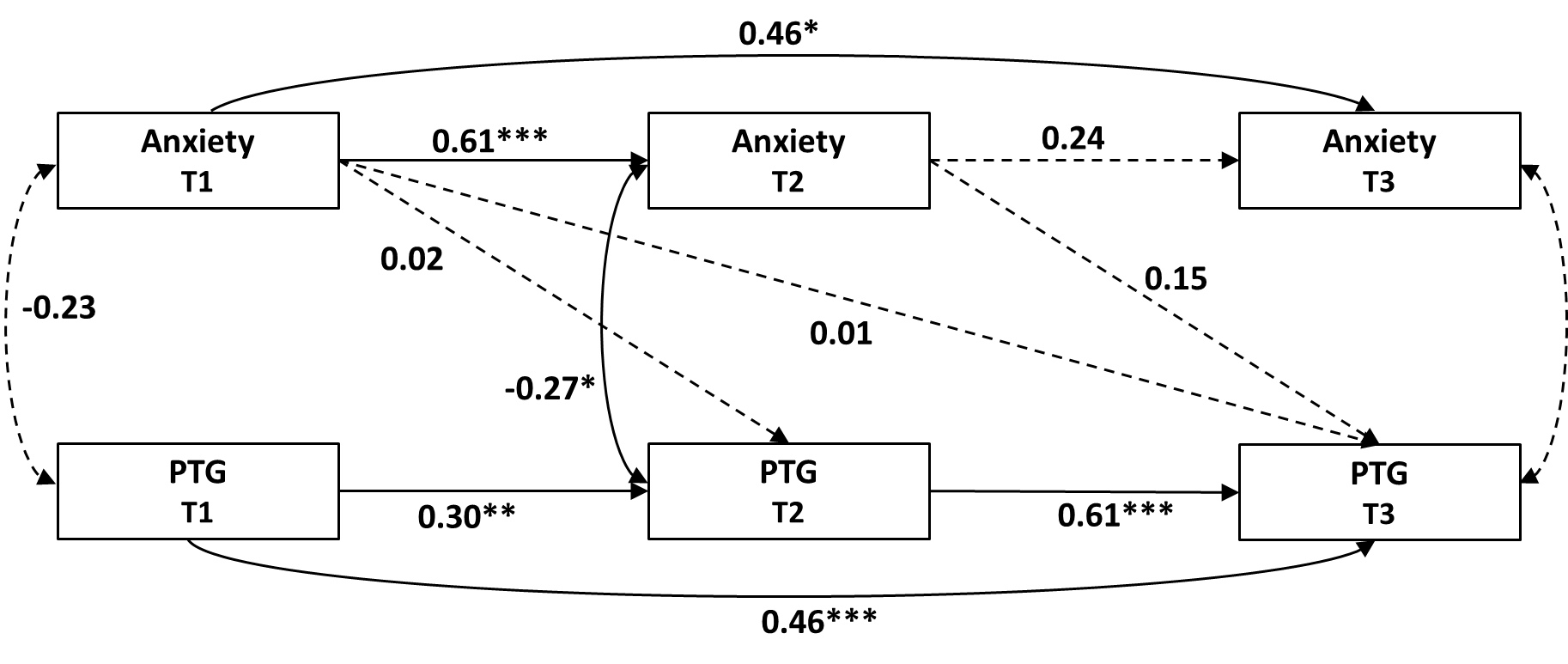
**

**Patients with higher financial burden**

**Figure S5.** The linear impact of anxiety on PTG, varied by levels of financial burden (healthcare cost ratio below 25% vs. 25% or higher). All paths shown are standardized regression coefficients. Solid lines indicate significant paths. Dashed lines indicate nonsignificant paths. PTG = posttraumatic growth

**p* < 0.05, ^**^*p* < 0.01, ^***^*p* < 0.001

**
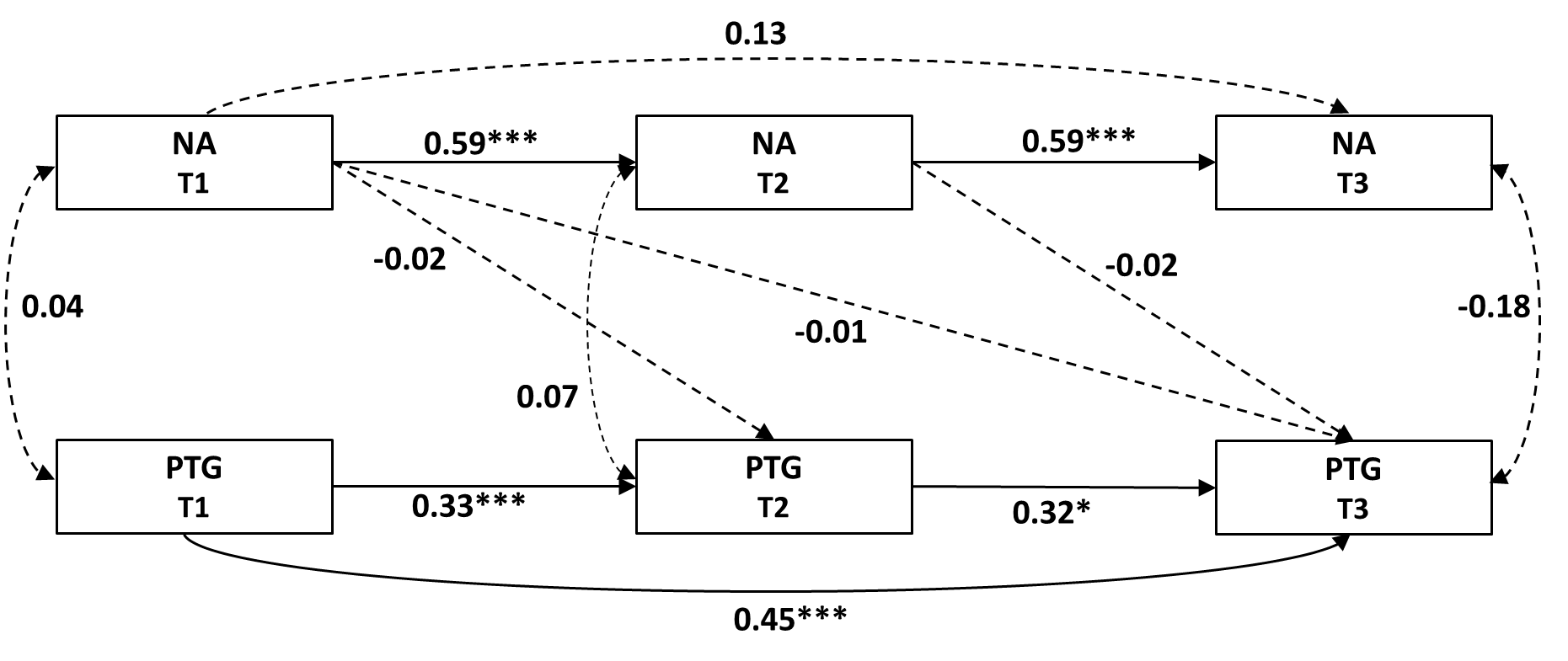
**

**Patients with younger age**

**
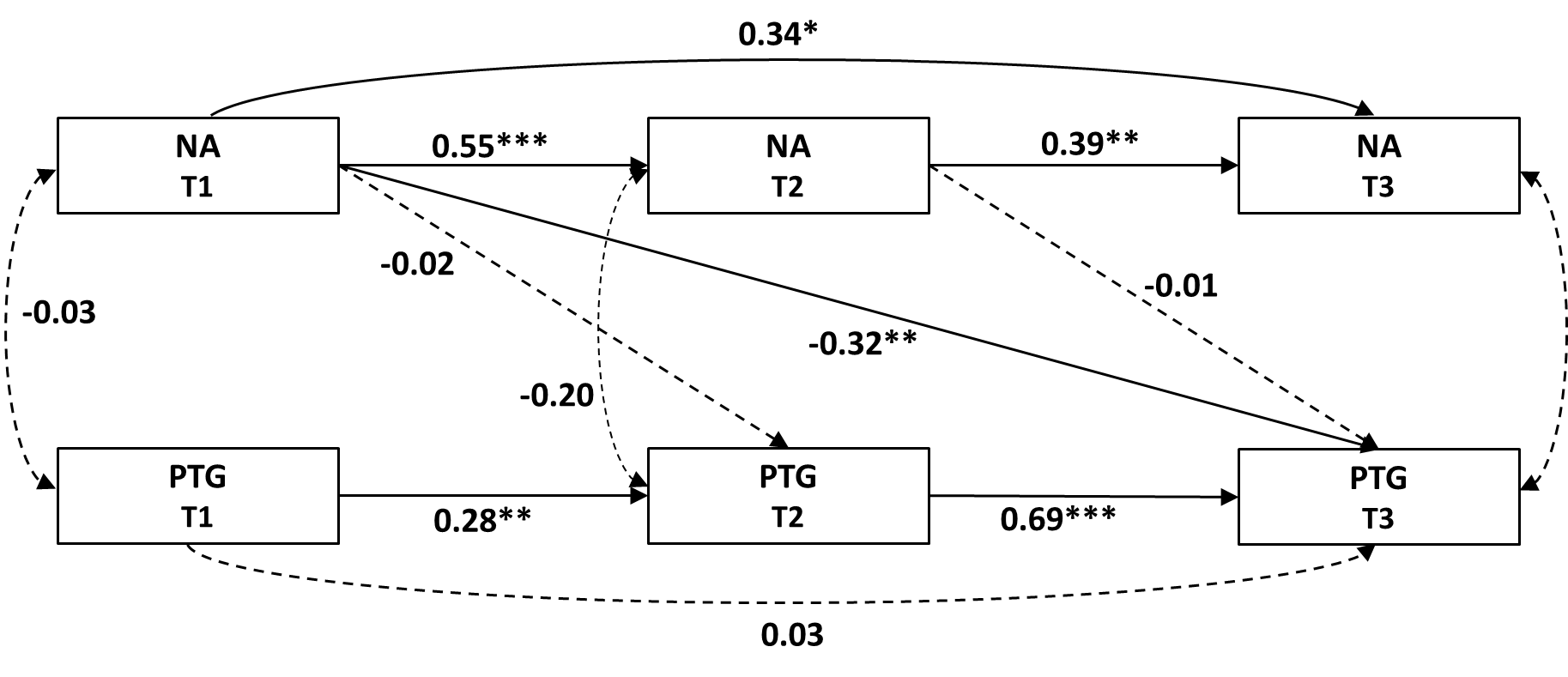
**

**Patients with older age**

**Figure S6.** The linear impact of negative affect on PTG, varied by patients’ age group (age ≤ 65 years vs. age > 65 years). All paths shown are standardized regression coefficients. Solid lines indicate significant paths. Dashed lines indicate nonsignificant paths. PTG = posttraumatic growth. NA = negative affect. **p* < 0.05, ^**^*p* < 0.01, ^***^*p* < 0.001

**
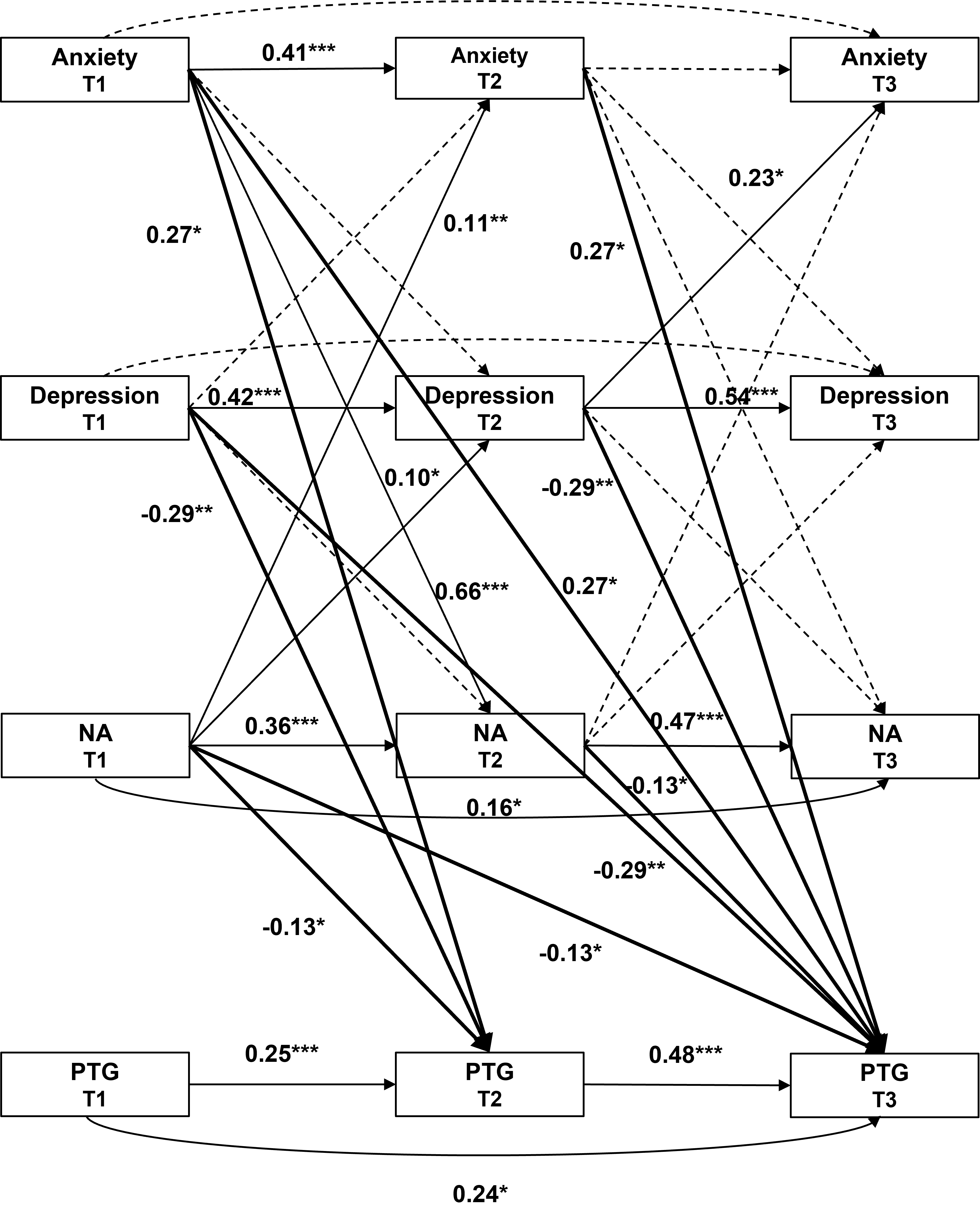
**

**Figure S7.** An alternative model that includes anxiety, depression, and NA as simultaneous predictors of PTG at subsequent waves. Cross-sectional covariances among the variables are omitted from the figure for conciseness. All paths shown are standardized regression coefficients. Solid lines indicate significant paths. Bold lines indicate cross-lagged paths between emotional distress and PTG. Dashed lines indicate nonsignificant paths. PTG = posttraumatic growth. NA = negative affect. **p* < 0.05, ^**^*p* < 0.01, ^***^*p* < 0.001
